# *In Silico* Feasibility Study of Carbon Ion Radiotherapy With Simultaneous Integrated Boost for Head and Neck Adenoid Cystic Carcinoma

**DOI:** 10.3389/fonc.2021.772580

**Published:** 2021-12-13

**Authors:** Edoardo Mastella, Silvia Molinelli, Giuseppe Magro, Stefania Russo, Maria Bonora, Sara Ronchi, Rossana Ingargiola, Alexandra D. Jensen, Mario Ciocca, Barbara Vischioni, Ester Orlandi

**Affiliations:** ^1^ Clinical Department, National Center for Oncological Hadrontherapy (CNAO), Pavia, Italy; ^2^ Department of Radiation Oncology, University Hospitals Gießen and Marburg (UKGM), Gießen, Germany; ^3^ FB20 (Medicine), Philipps University Marburg, Marburg, Germany

**Keywords:** simultaneous integrated boost (SIB), carbon ion radiation therapy (CIRT), head and neck cancer, adenoid cystic carcinoma (ACC), radiobiological models

## Abstract

**Purpose:**

In carbon ion radiotherapy (CIRT), a simultaneous integrated boost (SIB) approach has not been fully exploited so far. The feasibility of a CIRT-SIB strategy for head and neck adenoid cystic carcinoma (ACC) patients was investigated in order to improve treatment planning dose distributions.

**Methods and Materials:**

CIRT plans of 10 ACC patients treated at the National Center for Oncological Hadrontherapy (CNAO, Pavia, Italy) with sequential boost (SEQ) irradiation and prescription doses of 41.0 Gy [relative biological effectiveness (RBE)]/10 fractions to low-risk (LR) clinical target volume (CTV) plus 24.6 Gy(RBE)/6 fractions to the high-risk (HR) CTV were re-planned with two SIB dose levels to the LR-CTV, namely, 48.0 Gy(RBE) and 54.4 Gy(RBE). While planning with SIB, the HR-CTV coverage had higher priority, with fixed organ-at-risk dose constraints among the SIB and SEQ plans. The homogeneity and conformity indexes were selected for CTV coverage comparison. The biologically effective dose (BED) was calculated to compare the different fractionation schemes.

**Results:**

Comparable HR-CTV coverage was achieved with the treatment approaches, while superior conformality and homogeneity were obtained with the SIB technique in both CTVs. With the SEQ, SIB_48.0_, and SIB_54.4_, the LR-CTV median doses were respectively 50.3%, 11.9%, and 6.0% higher than the prescriptions. Significant reductions of the median and near-maximum BEDs were achieved with both SIB dose levels in the LR-CTV.

**Conclusions:**

The SIB approach resulted in highly conformal dose distributions with the reduction of the unintended dose to the LR-CTV. A prescription dose range for the LR-CTV will be clinically defined to offer tailored personalized treatments, according to the clinical and imaging characteristics of the patients.

## Introduction

Simultaneous integrated boost (SIB)–intensity-modulated radiation therapy (IMRT) has been one of the major technical photon-based RT innovations in the last 20 years.

For head and neck (HN) squamous cell carcinomas (SCCs), moderately accelerated IMRT techniques using SIB, usually at 2.12–2.2 and 1.7–2 Gy per fraction to the high-risk clinical target volume (HR-CTV) and low-risk (LR) CTV, respectively, in 30–33 fractions, with or without systemic therapy, have been largely adopted in clinical practice and within several prospective clinical trials, with similar results in terms of toxicity and oncologic outcome (1), whereas hypofractionated chemoradiation schedule with more than 2.4 Gy per fraction requires more caution to avoid severe toxicity (2).

For other non-SCC histologic tumor types historically known to be radioresistant, such as adenoid cystic carcinomas (ACCs) or other salivary gland cancers (SGCs), low linear energy transfer (LET) RT has not shown adequate local control, especially in the radical setting. Treatment outcome has recently improved with photon IMRT at doses of at least 70 Gy, with acceptable local control in patients with unresectable SGCs, especially in comparison with 3D techniques, since IMRT allows to better optimize the dose delivery, while reducing doses to the organs at risk (OARs) when escalating the dose to the target (3). ACC remains a major challenge for radiation oncologists, since it requires very high total doses to increase the probability to be cured. Moreover, its horseshoe shape is often anatomically complex, embracing or intersecting radiosensitive structures and following neural pathways (4). In recent years, strong evidence has been produced to support treatment with high-LET carbon ion RT (CIRT) for unresected ACC or after uncomplete surgical resection (5–11). Pencil beam scanning (PBS)-CIRT has entered the clinical practice for the treatment of ACC with beneficial effects on the outcome, in light of specific physical properties (allowing highly conformal dose distributions) and superior relative biological effectiveness (RBE) by at least a 1.5- to 3-fold factor in comparison with photons (5–11). Furthermore, CIRT has been shown to be effective even in more complex radioresistant scenarios, such as reirradiation of inoperable ACC (12) or of radiation-induced SGC (13), highlighting the need of further efforts to offer tailored personalized treatments, including particle therapy, to improve survival in cases of peculiar radioresistant phenotypes (14).

Until now, at our Institution (National Center for Oncological Hadrontherapy, CNAO, Pavia, Italy) (15), the PBS-CIRT standard approach for the treatment of ACC has been a sequential strategy consisting of a first phase of nine to 10 fractions to the LR-CTV followed by a second phase of six to seven fractions to the HR-CTV, with a unique nominal dose per fraction, according to the protocol adopted in Japan since 1997 (16). Japanese data of ACC CIRT treatments have recently been reported in a multicenter retrospective series with excellent results in terms of tumor local control and normal tissue toxicity (8). PBS-CIRT is usually delivered with a limited number of beams, typically two to three, achieving both high dose conformation and normal tissue sparing. However, with fixed-beam irradiation (horizontal and vertical directions at CNAO, where no isocentric gantry is available), it is very difficult to significantly change the beam arrangement between the two sequential phases; therefore part of the LR-CTV receives unintended dose from the beam paths of the boost phase.

In this paper, we investigated a SIB approach in comparison with our standard protocol for ACC patients treated with sequential boost (here called SEQ), in order to improve the actual dose distribution of the two target volumes currently delivered sequentially. While doing this, some aspects need to be taken into account, starting from the radiobiological model adopted in the treatment planning system (TPS) for RBE-weighted dose (DRBE) calculation. In fact, it is undoubtable that in CIRT, the radiobiological model has a strong impact on the dose delivered to the patient (17, 18). For ACC, a local effect model (LEM)-based (DRBE|LEM) prescription of 68.8 Gy(RBE) in 16 fractions to the HR-CTV was initially adopted to mimic the reference modified microdosimetric kinetic model (mMKM)-based (DRBE|MKM) prescription of 64 Gy(RBE) (19) adopted from HN clinical trial experience with CIRT in Japan. From the beginning of 2017, a slightly lower DRBE|LEM prescription of 65.6 Gy(RBE) has been used for patients at risk of major toxicity at our center. Since the majority of failures in our series were within the HR-CTV region due to the underdosage of the HR-CTV possibly due to the dose constraints to the OARs, we have hypothesized here that the total dose to the LR-CTV was enough to control microscopic tumor spread. In the view of a future clinical trial to implement the treatment of ACC patients, we aimed here to *in silico* investigate the feasibility of a SIB strategy instead of the standard SEQ protocol, comparing SIB plans with the original SEQ versions, in terms of target coverage, dose homogeneity, and dose conformality, in the two RBE abovementioned frames currently in clinical use worldwide. To our best knowledge, this is the first study that evaluates a SIB strategy for HN ACC patients treated with CIRT.

## Materials and Methods

### Patient Data

A retrospective dataset of 10 HN ACC patients was used: five ACC with parotid gland tumors and five originating from the minor salivary glands of paranasal sinuses/lacrimal glands were selected for the present analysis. These patients were consecutively treated at CNAO in a curative intent between October 2019 and September 2020 with CIRT in 16 fractions, delivered over 4 weeks according to our current SEQ strategy. The main patient clinical and treatment data are reported in **Table 1**.

**Table 1:** Clinical information of the adenoid cystic carcinoma patients.

Patient	Site	T N M*	LR-CTV		HR-CTV	
			Volume [cm^3^]	No. of beams	Volume [cm^3^]	No. of beams
P1	LG	cT4cN0	195.8	2	50.1	2
P2	PS	cT4aN0	618.2	3	301.9	2
P3	LG	cT4cN0	188.1	2	62.1	2
P4	LG	cT4bN0	174.3	2	50.0	2
P5	PS	cT4aNo	216.0	2	98.0	2
P6	PG	pT3cN0	189.1	2	60.2	1
P7	PG	cT3N1	342.4	2	179.9	2
P8	PG	cT4aN0	384.4	3	186.9	2
P9	PG	pT3cNo	238.8	2	71.5	2
P10	PG	pT3cN0	174.1	2	59.6	2

CTV, clinical target volume; LR, low risk; HR, high risk; LG, lacrimal glands; PS, paranasal sinuses; PG, parotid glands.

*Cancer staging according to the Union for International Cancer Control (UICC) TNM Classification of Malignant Tumors (8th Edition).

HR-CTV was defined as the macroscopic gross tumor volume (GTV) with a margin of 3–5 mm, whereas the LR-CTV included a margin of 2–5 mm around the HR-CTV and the perineural or compartmental spread of the disease. The mean LR-CTV was 272.1 ± 141.6 cm^3^, while the mean HR-CTV was 112.0 ± 84.1 cm^3^. Ethical approval by the institutional review board was obtained for this study, and all patients gave their written consent (CNAO study number OSS-26-2021).

### Sequential Boost Treatment Plans

Clinical delivered plans were optimized using RayStation-V8.1.2.5 TPS (RaySearch Laboratories AB, Stockholm, Sweden), with a prescribed dose of 4.1 Gy(RBE)|LEM/fraction. For the two sequential phases, beam arrangement and couch setup were selected depending on the target location. The first phase of the delivered treatment was planned with two beams for eight out of 10 patients and with three beams for the two other patients, while the SEQ (second phase) was planned with two beams for nine out of 10 patients and with one beam for the other patient (see **Table 1**). Our standard beam scanning parameters were used (20). Robust optimization (21) was performed using our standard settings: ± 3% range uncertainty, 2-mm isotropic isocenter shift. The LEM version I (22) was firstly used to calculate the DRBE|LEM with a α β ratio of 2 Gy. The prescribed dose of the first phase was 41.0 Gy(RBE) in 10 fractions to the median LR-CTV. A SEQ phase of 24.6 Gy(RBE)|LEM was then delivered in six more fractions to the median HR-CTV, reaching a total prescription DRBE|LEM of 65.6 Gy(RBE). In the plan optimization, the highest priority was sparing the brainstem and the optic pathways (at least the optic chiasm and the contra-lateral optic to preserve mono-lateral vision), with dose constraints derived from Dale et al. (23, 24). CTV coverage was thus increased as much as possible without exceeding the constraints of the OARs reported above. The main planning goals for volumes of interest are summarized in **Table 2**.

**Table 2:** Main planning goals for volumes of interest.

	Structures	Planning goals
CTVs	All CTVs	D95% ≥ 95% D98% ≥ 90%
	HR-CTV	D2% ≤ 103%
OARs	Brainstem	D1% ≤ 40 Gy(RBE)
	Optic pathways	D1% ≤ 45 Gy(RBE) D20% ≤ 37 Gy(RBE)
	Temporal and frontal lobes	D2cc < 54 Gy(RBE)

Note. CTV, clinical target volume; HR, high risk; OAR, organ at risk; D%, dose to % of the volume of interest.

### Simultaneous Integrated Boost Treatment Plans

The delivered SEQ treatment plans were compared with two SIB fractionation schemes with different low dose levels: 54.4 Gy(RBE) (“SIB_54.4_”) and 48.0 Gy(RBE) (“SIB_48.0_”) to the median LR-CTV in 16 fractions and a high dose level of 65.6 Gy(RBE) to the HR-CTV. Thus, nominal dose per fraction to LR-CTV was 3.4 Gy (RBE) in SIB_54.4_ and 3.0 Gy(RBE) in SIB_48.0_. These prescription doses were investigated as they resulted in a nominal LR-CTV biologically effective dose (BED) comparable with the SEQ approach when using the different RBE models (see the section *Treatment Plan Evaluation*). The median LR-CTV was defined as the volume of the LR-CTV minus HR-CTV. The same beam arrangements and scanning parameters of the first phase of the SEQ plan were used, together with the same robustness settings. In the SIB plans, priority was given to the HR-CTV coverage to reproduce a dose–volume histogram (DVH) similar to the SEQ keeping fixed the same OAR dose constraints. A total of 20 SIB plans were optimized by the same experienced planner.

### Modified Microdosimetric Kinetic Model Dose Translation

Being our clinical protocols derived from the CIRT protocols tested in clinical trials in Japan (17) and thus our approach ensued from the mMKM-based experience (19), all SEQ and SIB absorbed dose distributions were translated into mMKM doses using the V6.99 research RayStation version, previously commissioned for our beamline (19). A total of 40 plans were recalculated.

### Treatment Plan Evaluation

The SEQ and SIB techniques were firstly compared in terms of DVH-based metrics. In particular, the near-minimum (D98%), the median (D50%), and the near-maximum (D2%) doses were chosen as dose-summarizing parameters (DSPs) for CTV coverage evaluation, as recommended by the International Commission on Radiation Units and Measurements (ICRU) 78 (25). The DSPs were normalized to the corresponding prescriptions. For mMKM doses, the nominal prescriptions were obtained through the relations calculated in Fossati et al. and Wang et al. (17, 26). The doses/fractions of 4.1, 3.4, and 3.0 Gy(RBE)|LEM corresponded to 3.5, 2.6, and 2.2 Gy(RBE)|mMKM, respectively. Dose evaluators for the LR-CTV were referred to the LR-CTV minus the HR-CTV.

The homogeneity index (HI) and conformity index (CI) of the CTVs were evaluated as recommended by the ICRU 93 (27) and were calculated for the whole treatment similarly to Orlandi et al. (28):


(1) $$HI=\frac{D2\%-D98\%}{Dprescription}$$

(2) $$CI=\frac{TV_{D98\%}}{CTV}$$

where TV is the total volume encompassed by the D98%.

Differences of the DSP were calculated as SIB minus SEQ plans:


(2) $$\Delta DSP=DSP_{SIB}-DSP_{SEQ}$$

Afterwards, we calculated the BED to compare the different fractionation schemes similarly to Kawashiro et al. (29):


(3) $$BED_{\alpha /\beta }=nd\left(1+\frac{d}{\alpha /\beta }\right)$$

where α and β are coefficients of the linear-quadratic model, n is the number of fractions, and d is the dose per fraction. For the SEQ approach, the total BED was the sum of the single-phase BEDs. We assumed a α/β ratio of 3 for ACC. The SEQ prescription of 41.0 Gy(RBE)|LEM in 10 fractions for the LR-CTV corresponded to a BED of 97.0 Gy(RBE), while the SIB levels of 48.0 and 54.4 Gy(RBE)|LEM in 16 fractions corresponded to a BED of 96.0 Gy(RBE) and 116.1 Gy(RBE). When translating to mMKM, the nominal BEDs of the first phase of the SEQ, SIB_48.0_, and SIB_54.4_ were respectively 75.8, 61.0, and 77.7Gy(RBE).

The equivalent dose in 2-Gy(RBE) fractions (EQD2) was also calculated as


(4) $$EQD2_{\alpha /\beta }=\frac{BED_{\alpha /\beta }}{1+\frac{2}{\alpha /\beta }}$$

Statistical significance of the dosimetric parameters was assessed with the non-parameter Wilcoxon signed-rank test, using MATLAB-based software (version R2018a, The MathWorks Inc., Natick, MA, USA). A significance level of 5% was chosen (*p*-value <0.05). In the *post-hoc* analysis, the significance level was corrected using the Bonferroni method based on the number of multiple tests conducted (n = 5 for the DSPs; n = 3 for the BEDs).

## Results

**Figure 1:**
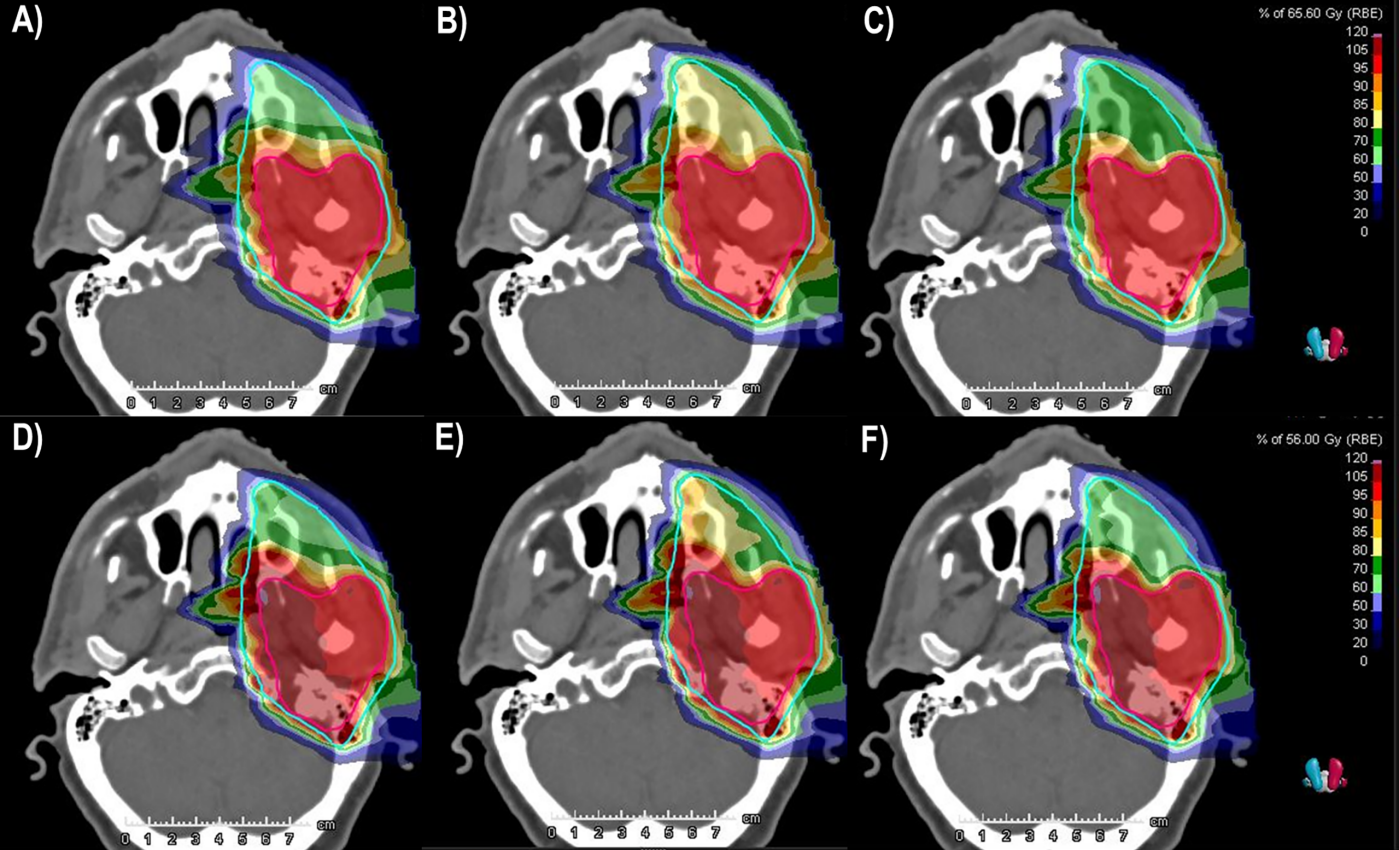
LEM and mMKM dose distributions calculated for patient P6. The top (bottom) panel corresponds to LEM (mMKM) doses: **(A, D)** SEQ approach. **(B, E)** SIB_54.4_. **(C, F)** SIB_48.0_. The HR-CTV and LR-CTV are delineated in purple and light blue, respectively. The isodoses were normalized to the HR-CTV prescriptions. LEM, local effect model; mMKM, modified microdosimetric kinetic model; SEQ, sequential boost; SIB, simultaneous integrated boost; HR-CTV, high-risk clinical target volume; LR-CTV, low-risk clinical target volume.

**Figure 1** shows the dose distributions of a representative case (P6) obtained with the SEQ and SIB approaches: the top panel corresponds to LEM doses and the bottom panel to mMKM doses.

**Figure 2:**
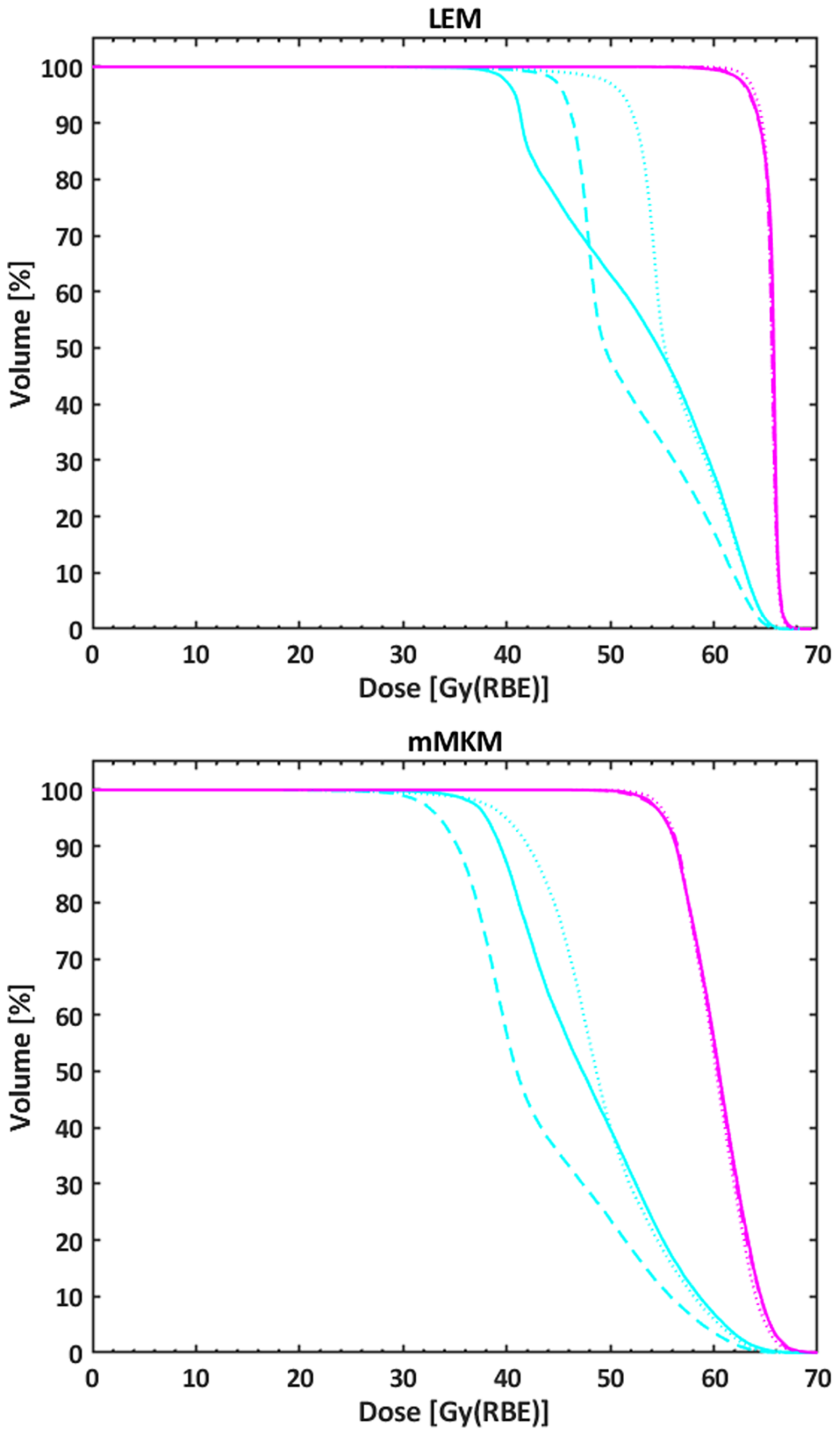
DVHs of the HR-CTV (purple line) and the LR-CTV (light blue line) of P6 achieved with the SEQ (solid line), the SIB_54.4_ (dotted line), and the SIB_48.0_ (dashed line) approaches. The top (bottom) panel corresponds to LEM (mMKM) doses. DVH, dose–volume histogram; HR-CTV, high-risk clinical target volume; LR-CTV, low-risk clinical target volume; SEQ, sequential boost; SIB, simultaneous integrated boost; LEM, local effect model; mMKM, modified microdosimetric kinetic model.

**Figure 2** shows the DVHs of the HR-CTV and the LR-CTV of P6 achieved with the SEQ and SIB techniques.

The mean values of the DSP are reported in **Table 3**, obtained with both the LEM and the mMKM for the different treatment approaches. The values were normalized to the corresponding prescriptions.

**Table 3:** Mean values ( ± 1 standard deviation) of the target coverage achieved with the three treatment approaches, reported for both radiobiological calculation models.

		HR-CTV					LR-CTV				
		D98%	D50%	D2%	HI	CI	D98%	D50%	D2%	HI	CI
		[%]	[%]	[%]			[%]	[%]	[%]		
LEM	SEQ	97.2 ± 2.1	100.0 ± 0.0	101.5 ± 0.8	0.04 ± 0.03	1.38 ± 0.23	100.0 ± 1.5	150.3 ± 11.9	166.3 ± 8.4	0.66 ± 0.08	1.56 ± 0.13
	SIB_54.4_	96.2 ± 2.3	100.0 ± 0.0	101.7 ± 0.6	0.05 ± 0.03	**1.06 ± 0.07**	**91.2 ± 3.3**	**106.0 ± 5.7**	**120.7 ± 1.0**	**0.30 ± 0.03**	**1.19 ± 0.13**
	SIB_48.0_	95.4 ± 2.2	100.0 ± 0.0	102.0 ± 0.5	0.07 ± 0.03	**0.99 ± 0.04**	**92.2 ± 2.3**	**111.9 ± 10.6**	**136.6 ± 1.6**	**0.44 ± 0.02**	**1.33 ± 0.12**
mMKM	SEQ	98.7 ± 2.2	106.6 ± 1.8	117.6 ± 4.7	0.19 ± 0.06	1.78 ± 0.36	105.4 ± 2.8	153.8 ± 20.9	191.3 ± 11.2	0.86 ± 0.11	1.50 ± 0.16
	SIB_54.4_	97.7 ± 2.5	106.2 ± 1.7	116.4 ± 2.7	0.19 ± 0.04	**1.39 ± 0.19**	**91.5 ± 4.5**	**118.8 ± 11.9**	**149.4 ± 3.8**	**0.58 ± 0.05**	**1.39 ± 0.13**
	SIB_48.0_	96.9 ± 2.1	106.6 ± 1.7	116.9 ± 2.2	0.20 ± 0.03	**1.27 ± 0.15**	**90.0 ± 3.5**	**122.8 ± 19.9**	**175.2 ± 4.3**	0.85 ± 0.05	1.47 ± 0.11

The values were normalized to the corresponding prescriptions. Statistically significant differences between the SEQ and SIB approaches are shown in bold (p-values are statistically significant after Bonferroni correction of five tests, p < 0.01).

CTV, clinical target volume; HR, high risk; LR, low risk; D%, dose to % of the CTV; HI, homogeneity index; CI, conformity index; LEM, local effect model; mMKM, modified microdosimetric kinetic model; SEQ, sequential boost; SIB, simultaneous integrated boost.

All optimized plans concern the OAR dose constraints reported in **Table 2**.

After Bonferroni correction of five tests, comparable HR-CTV coverage was found between LEM-based SEQ and SIB plans (SIB_48.0_: *p* = 0.014 for both D98% and D2%; SIB_54.4_: *p* = 0.13 and *p* = 0.38 for D98% and D2%). On average, a very slight decrease of −1.8% was observed in the D98% with the SIB_48.0_, which was on average compliant with the optimization goal (D95% ≥ 95% and D2% ≤ 103% of the prescribed dose). HR-CTV dose inhomogeneity slightly increased with the SIB, while the HI values of the LR-CTV decreased significantly for both SIB dose levels. With the SEQ approach, 50% of the LR-CTV received on average a dose 50.3% higher than the prescription, while differences between LR-CTV median and prescribed doses were reduced to 11.9% and 6.0% for SIB_48.0_ and SIB_54.4_, respectively.

Dose inhomogeneity was slightly higher for ACC of paranasal sinuses/lacrimal glands than parotid glands cases due to the target location closer to critical structures. In particular, for ACC of paranasal sinuses/lacrimal glands, the average HI values of the HR(LR)-CTV were 0.06 ± 0.02 (0.68 ± 0.09), 0.07 ± 0.03 (0.30 ± 0.05), and 0.08 ± 0.02 (0.45 ± 0.03) for the SEQ, SIB_54.4_, and SIB_48.0_, respectively (see **Supplementary Table S1**).

The CI values of both CTVs were significantly reduced with the SIB, indicating a better conformality of this technique with respect to the SEQ.

As expected, DRBE became more inhomogeneous when translating into mMKM doses (19), with an increase of the HI in both CTVs for all treatment approaches (see **Table 3**). However, mMKM ΔDSP between the SIB and SEQ techniques decreased for HR-CTV, with respect to LEM, with comparable HR-CTV coverage for all optimization approaches. In particular, the *p*-values of the SIB_48.0_ and SIB_54.4_ were respectively 0.065 (0.87) and 0.39 (0.77) for D98% (D2%).

The mMKM dose distributions confirmed the superior conformality of the SIB technique in both CTVs. When considering the LR-CTV, the dose distributions of the SIB were also more homogeneous, with the best homogeneity and conformality obtained with the SIB_54.4_.

All the dose distributions of the paranasal sinuses/lacrimal glands group were slightly more inhomogeneous than the parotid gland group, as highlighted with the LEM. For these patients, the average HI values of the HR(LR)-CTV were 0.21 ± 0.07 (0.92 ± 0.10), 0.19 ± 0.05 (0.60 ± 0.07), and 0.20 ± 0.04 (0.87 ± 0.06) for the SEQ, SIB_54.4_, and SIB_48.0_, respectively (see **Supplementary Table S2**).

The mean values of the BED calculated with both RBE models are summarized in **Table 4**.

**Table 4:** Mean values ( ± 1 standard deviation) of the biologically effective dose (BED) obtained with the three treatment approaches, reported for both radiobiological calculation models.

		HR-CTV			LR-CTV		
		BED (D98%)	BED (D50%)	BED (D2%)	BED (D98%)	BED (D50%)	BED (D2%)
		[Gy(RBE)]	[Gy(RBE)]	[Gy(RBE)]	[Gy(RBE)]	[Gy(RBE)]	[Gy(RBE)]
LEM	SEQ	148.4 ± 5.3	155.3 ± 0.0	158.9 ± 2.1	87.6 ± 5.5	135.6 ± 11.0	156.8 ± 1.3
	SIB_54.4_	146.1 ± 5.1	155.3 ± 0.0	159.4 ± 1.4	**100.9 ± 5.5**	**127.1 ± 10.2**	155.5 ± 2.1
	SIB_48.0_	**144.1 ± 5.1**	155.3 ± 0.0	**160.2 ± 1.3**	85.0 ± 3.1	**114.4 ± 16.9**	155.1 ± 2.9
mMKM	SEQ	119.0 ± 4.1	134.0 ± 3.4	156.3 ± 10.0	71.1 ± 4.8	112.4 ± 14.2	152.5 ± 6.3
	SIB_54.4_	117.1 ± 4.6	133.2 ± 3.3	153.7 ± 5.5	68.4 ± 4.8	**100.8 ± 16.3**	**142.7 ± 5.6**
	SIB_48.0_	115.7 ± 3.8	134.0 ± 3.4	154.7 ± 4.5	**52.6 ± 2.8**	**83.1 ± 22.0**	**141.0 ± 5.3**

Statistically significant differences between the SEQ and SIB approaches are shown in bold (p-values are statistically significant after Bonferroni correction of three tests, p < 0.017).

CTV, clinical target volume; HR, high risk; LR, low risk; LEM, local effect model; mMKM, modified microdosimetric kinetic model; SEQ, sequential boost; SIB, simultaneous integrated boost.

After Bonferroni correction of three tests, a significant decrease of the BED(D98%)|LEM was found with the SIB_48.0_ in the HR-CTV (*p* = 0.014), together with slight increase of the BED(D2%)|LEM (*p* = 0.01). When translated into mMKM doses, these values were comparable: *p* = 0.065 for BED(D98%)|mMKM and *p* = 0.92 for BED(D2%)|mMKM. When considering the LR-CTV, the BEDs calculated for the median doses were significantly lower for both SIB dose levels and RBE models with respect to the SEQ plans (*p* = 0.002).

With the LEM, the near-minimum BED(D98%)|LEM was comparable between the SEQ and SIB_48.0_ (*p* = 0.19), while a statistically significant increase was found for SIB_54.4_ plans (*p* = 0.002).

Opposite to the LEM, with the mMKM, a significant difference was found in the BED(D98%)|mMKM of the LR-CTV for the SIB_48.0_ (*p* = 0.002), while the BED(D98%)|mMKM of the SIB_54.4_ was comparable with the SEQ (*p* = 0.13).

Statistically significant decreases of the BED(D2%)|mMKM were observed for both SIB dose levels (*p* = 0.002), with a reduction of about 10 Gy(RBE).

The mean values of the EQD2 calculated for the three treatment approaches and both RBE models are reported in **Supplementary Table S3**.

## Discussion

Our CIRT prescription doses were defined with the aim of reproducing the National Institute of Radiological Sciences (NIRS) clinical results, with long follow-up available that provides reassuring conclusions on long-term toxicity. The RBE model currently in clinical use at NIRS is the mMKM. At the time of this study, no commercial TPS provided clinical mMKM doses, and the European experience was primarily based on the LEM model. Therefore, using the same fractionation schemes of the NIRS, we corrected the LEM-based prescription doses for the RBE dependence as described in Fossati et al. for simple geometric configuration (17). The practically feasible way to deliver treatment plans as close as possible, from a clinical point of view, to NIRS practice was to minimize the physical dose differences between the two RBE models. Conversion factors ranging from 1.04 to 1.15 were initially adopted to translate DRBE|mMKM into the DRBE|LEM prescription dose levels that deliver the closest absorbed dose. Prudentially, no correction was applied to OAR constraints. Prescription dose conversion factors increase as the mMKM prescribed dose per fraction decreases. A new approach combining Monte Carlo simulations was then implemented for real patient data in order to further verify the consistency of our conversion factors (18). Our group has recently investigated the impact of RBE modelling on treatment outcomes of a series of 78 ACC patients treated between 2013 and 2016 with the SEQ approach (19). The study highlighted that the majority of the recurrences could be explained by inadequate HR-CTV coverage, mainly due to conservative dose constraints to the OARs. New constraints for the brainstem and optic pathways were then defined to improve target coverage and dose homogeneity in treatment planning, considering the uncertainties in dose biological modelling translation of Japanese protocols to LEM (23, 24). Until now, no increment in normal tissue complications has been observed in our treated patients with the adoption of new dose limits for OARs (30). In this study, with these new OAR constraints, we have evaluated for 10 ACC patients the dose distribution of a SIB treatment in comparison with the actual clinical scenario of SEQ volume, with the aim of improving the tumor dose conformation while reducing the unintended dose to the LR volume and related probability of complications.

In addition, the SIB technique is known to be a more practical and efficient treatment approach (31). A single optimization process is needed, thus facilitating the optimizer in the search for the optimal solution balancing target coverage and OAR sparing over the whole treatment. Moreover, the clinical workflow would benefit from the SIB strategy, e.g., in the patient-specific quality assurance process or in case of treatment adaptation.

Only Kawashiro et al. (29) investigated a SIB treatment using PBS-CIRT, although it is the more convenient procedure, to our best knowledge. In their *in silico* study, the authors evaluated a SIB strategy for pancreatic cancer to increase tumor dose while sparing OARs. Their analysis suggested the feasibility of dose escalation using the SIB satisfying the dose constraints for OARs, underlying that the SIB technique has the potential to deliver higher doses to the tumor, without substantially increasing the dose to OARs. Of course, their results should be confirmed in future patient clinical trials.

We investigated two different SIB prescriptions that resulted in a nominal LR-CTV BED comparable with the SEQ approach when using the different RBE models. As recently underlined in Molinelli et al. (32), in CIRT, a combined multimodel RBE-based optimization could play a key role in the enhancement of the therapeutic ratio for radioresistant tumors. In particular, the lower SIB dose level, i.e., DRBE|LEM prescription of 48.0 Gy(RBE), resulted in a nominal LR-CTV BED comparable with the LEM-based SEQ approach: BED(SIB_48.0_)|LEM = 96.0 Gy(RBE) *vs*. BED(SEQ)|LEM = 97.0 Gy(RBE). As the prescription dose conversion factors to correct for differences in the RBE model increase for lower doses per fraction (17, 18), we also examined a higher SIB dose level, i.e., 54.4 Gy(RBE), which maintains a similar mMKM-based LR-CTV nominal BED of the SEQ plan: BED(SIB_54.4_)|mMKM = 77.7 Gy(RBE) *vs*. BED(SEQ)|mMKM = 75.8 Gy(RBE).

The sample size of this work was small for practical reasons. However, most of the studies that compare dosimetric data in RT include between 10 and a few hundred patients, as reported by Chaikh et al. (33). Of course, a greater number of patients would be needed to strengthen our results. A limitation of the statistical analysis could be that a paired test was adopted, thus increasing the probability of finding statistical significance even when the actual difference is low in absolute values. However, as the Wilcoxon signed-rank test does not require a normal distribution and does not consider the size of the difference, it particularly fits for RT dosimetric comparison, since the data are “naturally” paired (33). Another limitation could be that using multiple summarizing parameters could inflate the false-positive rate. Therefore, the Bonferroni correction was computed to avoid inflating type 1 error probability (33).

In the plan optimization, with the SIB_48.0_, we obtained a slightly lower D98%|LEM of the HR-CTV even respecting the planning goals, probably due to the high dose gradient between the CTV prescription dose levels. A superior conformality was obtained with the SIB technique in both CTVs, together with an improved homogeneity and a significant reduction in the biological median and near-maximum doses to the LR-CTV. The SIB_54.4_ achieved the best homogeneity and conformality with both RBE models.

Plan robustness may be an issue in extremely modulated plans, especially in the paranasal sinuses. Our clinical protocol foresees periodic re-evaluation CT scans every other week or whenever deemed necessary according to patients’ status and daily imaging.

As expected, a higher BED to the 98% of the LR-CTV was obtained with the SIB_54.4_ when using the LEM, with an average value comparable with photon-based RT with a prescription dose of 60 Gy (34). On the other hand, this value turned out to be comparable with that of the SEQ approach when translating to mMKM. Predictably, with the SIB approach, the LR-CTV was more sensitive to the translation between LEM and mMKM (see **Figure 2** and **Table 4**) as a result of the lower prescribed dose per fraction (e.g., higher conversion factors between the models).

The issue of the effective clinical dose to the LR volume and perineural spread for ACC tumors has been deeply investigated in photon-based RT (34). When considering both BED and EQD2 values of the SIB_48.0_ and SIB_54.4_, both dose levels would be equally acceptable when treating ACC tumors, depending on the prognostic factors.

In consideration of the near-minimum BED obtained with the mMKM and of the superior dose distributions achieved with the SIB_54.4_, we believe that for future clinical trials of CIRT in ACC patients, the 54.4 Gy(RBE)|LEM dose level could assure adequate tumor control in case of positive margin along the nerve, whereas the 48.0 Gy(RBE)|LEM dose level could have adequate efficacy in case of elective perineural irradiation or microscopic focal intratumor perineural invasion (PNI), maintaining a low toxicity profile.

Furthermore, concerning the fractionation scheme, adopting the SIB strategy instead of the sequential approach would allow to maintain a high dose/fraction (4.1 Gy(RBE)|LEM/fraction) for the more radioresistant GTV in the HR-CTV, lowering the fraction dose for the microscopic disease (3.0–3.4 Gy(RBE)|LEM/fraction) embedded within the normal OARs of the LR-CTV.

A limitation of this study is that a retrospective dataset was used and a greater number of patients would strengthen these results. Only five ACC of paranasal sinuses/lacrimal glands were selected where the HR-CTV is closer/adjacent to critical organs (e.g., brainstem and optic pathways). However, as the HR-CTV prescription dose was the same between the two treatment approaches, the OAR dose constraints of the SIB plans corresponded to the total SEQ approach and for this reason were not reported in this analysis.

## Conclusion

In conclusion, this study investigated the SIB feasibility for CIRT in HN ACC for both the LEM and mMKM radiobiological models. The advantage of the SIB approach would be a better dose conformality with the reduction of the unintended dose to the LR-CTV in comparison with the standard strategy of SEQ. A prescription dose range for the LR-CTV will be clinically defined to offer tailored personalized treatments, according to the clinical and imaging characteristics of the patients.

## Data Availability Statement

The original contributions presented in the study are included in the article/**Supplementary Material**. Further inquiries can be directed to the corresponding author.

## Ethics Statement

The studies involving human participants were reviewed and approved by the institutional review board (CNAO study number OSS-26-2021). The patients/participants provided their written informed consent to participate in this study. Written informed consent was obtained from the individual(s) for the publication of any potentially identifiable images or data included in this article.

## Author Contributions

EM, SM, and EO contributed to the conception and design of the study. EM, MB, and BV organized the database. EM and GM performed the statistical analysis. EM and EO wrote the first draft of the manuscript. SM and BV wrote sections of the manuscript. EM, SM, GM, SRu, MB, SRo, RI, AJ, MC, BV, and EO contributed to the manuscript revision and read and approved the submitted version. All authors contributed to the article and approved the submitted version.

## Conflict of Interest

The authors declare that the research was conducted in the absence of any commercial or financial relationships that could be construed as a potential conflict of interest.

## Publisher’s Note

All claims expressed in this article are solely those of the authors and do not necessarily represent those of their affiliated organizations, or those of the publisher, the editors and the reviewers. Any product that may be evaluated in this article, or claim that may be made by its manufacturer, is not guaranteed or endorsed by the publisher.
