# Supplementary material for: In Silico Feasibility Study of Carbon Ion Radiotherapy With Simultaneous Integrated Boost for Head and Neck Adenoid Cystic Carcinoma
Source: Front Oncol. 2021 Dec 13;11:772580. doi: 10.3389/fonc.2021.772580 (PMC8710479; doi:10.3389/fonc.2021.772580)
Supplement: Supplementary file 1 [file Table_1.docx]

**Supplementary Table S1.** Target coverage achieved with the three treatment approaches, reported for the local effect model. The values were normalized to the corresponding prescriptions.

|  |  | HR-CTV | | | | | LR-CTV | | | | |
| --- | --- | --- | --- | --- | --- | --- | --- | --- | --- | --- | --- |
|  |  | D98% | D50% | D2% | HI | CI | D98% | D50% | D2% | HI | CI |
|  |  | [%] | [%] | [%] |  |  | [%] | [%] | [%] |  |  |
| SEQ | P1 | 93.4 | 100.0 | 101.5 | 0.08 | 1.48 | 101.2 | 147.1 | 162.2 | 0.61 | 1.51 |
|  | P2 | 98.3 | 100.0 | 101.2 | 0.03 | 1.27 | 100.2 | 159.0 | 161.7 | 0.61 | 1.36 |
|  | P3 | 96.2 | 100.0 | 103.4 | 0.07 | 1.38 | 99.3 | 148.3 | 161.2 | 0.62 | 1.75 |
|  | P4 | 94.8 | 100.0 | 101.2 | 0.06 | 1.67 | 100.5 | 162.6 | 178.9 | 0.78 | 1.56 |
|  | P5 | 97.0 | 100.0 | 101.8 | 0.05 | 1.24 | 101.6 | 166.1 | 178.6 | 0.77 | 1.76 |
|  | P6 | 99.4 | 100.0 | 101.7 | 0.02 | 1.41 | 97.6 | 129.8 | 161.5 | 0.64 | 1.56 |
|  | P7 | 99.4 | 100.0 | 100.6 | 0.01 | 1.20 | 100.2 | 145.1 | 160.5 | 0.60 | 1.62 |
|  | P8 | 95.4 | 100.0 | 101.6 | 0.06 | 0.94 | 97.3 | 133.9 | 159.3 | 0.62 | 1.35 |
|  | P9 | 99.2 | 100.0 | 100.5 | 0.01 | 1.78 | 100.5 | 156.4 | 178.0 | 0.78 | 1.47 |
|  | P10 | 98.5 | 100.0 | 101.2 | 0.03 | 1.49 | 101.2 | 155.1 | 161.3 | 0.60 | 1.64 |
| SIB_54.4_ | P1 | 96.8 | 100.0 | 101.5 | 0.05 | 1.02 | 89.0 | 101.7 | 122.1 | 0.33 | 1.03 |
|  | P2 | 96.6 | 100.0 | 101.5 | 0.05 | 1.01 | 94.5 | 117.6 | 122.1 | 0.28 | 1.13 |
|  | P3 | 95.7 | 100.0 | 102.6 | 0.07 | 0.97 | 93.8 | 101.8 | 119.4 | 0.26 | 1.21 |
|  | P4 | 90.5 | 100.0 | 102.4 | 0.12 | 1.19 | 84.6 | 104.2 | 120.8 | 0.36 | 1.14 |
|  | P5 | 95.1 | 100.0 | 102.5 | 0.07 | 1.11 | 94.3 | 112.3 | 121.3 | 0.27 | 1.42 |
|  | P6 | 97.6 | 100.0 | 101.1 | 0.04 | 1.07 | 91.2 | 103.5 | 119.7 | 0.28 | 1.03 |
|  | P7 | 99.3 | 100.0 | 101.1 | 0.02 | 1.04 | 94.1 | 104.0 | 120.4 | 0.26 | 1.32 |
|  | P8 | 96.3 | 100.0 | 101.6 | 0.05 | 0.97 | 89.9 | 101.7 | 119.9 | 0.30 | 1.08 |
|  | P9 | 97.0 | 100.0 | 101.4 | 0.04 | 1.12 | 87.9 | 101.5 | 119.9 | 0.32 | 1.32 |
|  | P10 | 97.1 | 100.0 | 101.2 | 0.04 | 1.11 | 92.5 | 111.4 | 121.7 | 0.29 | 1.20 |
| SIB_48.0_ | P1 | 95.7 | 100.0 | 102.1 | 0.06 | 0.98 | 90.8 | 104.0 | 139.0 | 0.48 | 1.23 |
|  | P2 | 95.6 | 100.0 | 102.0 | 0.06 | 0.98 | 95.6 | 132.5 | 139.0 | 0.43 | 1.33 |
|  | P3 | 94.7 | 100.0 | 103.0 | 0.08 | 0.95 | 91.3 | 104.6 | 135.2 | 0.44 | 1.29 |
|  | P4 | 90.5 | 100.0 | 102.3 | 0.12 | 1.08 | 87.3 | 110.6 | 136.0 | 0.49 | 1.39 |
|  | P5 | 94.4 | 100.0 | 102.5 | 0.08 | 0.98 | 93.5 | 124.8 | 136.9 | 0.43 | 1.60 |
|  | P6 | 96.6 | 100.0 | 101.8 | 0.05 | 1.02 | 91.0 | 109.0 | 135.2 | 0.44 | 1.22 |
|  | P7 | 99.2 | 100.0 | 101.4 | 0.02 | 0.98 | 94.0 | 107.7 | 136.3 | 0.42 | 1.43 |
|  | P8 | 95.0 | 100.0 | 101.8 | 0.07 | 0.92 | 92.3 | 103.1 | 135.0 | 0.43 | 1.15 |
|  | P9 | 96.0 | 100.0 | 101.5 | 0.05 | 1.01 | 92.5 | 101.7 | 135.0 | 0.43 | 1.35 |
|  | P10 | 95.9 | 100.0 | 101.5 | 0.06 | 1.01 | 93.3 | 121.5 | 138.1 | 0.45 | 1.29 |

**Abbreviations:** CTV = clinical target volume; HR = high risk; LR = low risk; D% = dose to % of the CTV; HI = homogeneity index; CI = conformity index; SEQ = sequential boost; SIB = simultaneous integrated boost; P = patient.

**Supplementary Table S2.** Target coverage obtained after modified microdosimetric kinetic model dose translation, reported for the three treatment approaches. The values were normalized to the corresponding prescriptions.

|  |  | HR-CTV | | | | | LR-CTV | | | | |
| --- | --- | --- | --- | --- | --- | --- | --- | --- | --- | --- | --- |
|  |  | D98% | D50% | D2% | HI | CI | D98% | D50% | D2% | HI | CI |
|  |  | [%] | [%] | [%] |  |  | [%] | [%] | [%] |  |  |
| SEQ | P1 | 95.2 | 105.9 | 116.6 | 0.21 | 2.04 | 106.9 | 136.3 | 188.6 | 0.82 | 1.19 |
|  | P2 | 97.3 | 103.2 | 113.9 | 0.17 | 1.40 | 102.0 | 148.3 | 184.6 | 0.83 | 1.32 |
|  | P3 | 97.5 | 109.3 | 130.0 | 0.33 | 2.03 | 101.4 | 153.1 | 196.6 | 0.95 | 1.52 |
|  | P4 | 99.5 | 106.6 | 116.1 | 0.17 | 2.36 | 102.5 | 169.5 | 206.3 | 1.04 | 1.55 |
|  | P5 | 101.3 | 108.2 | 118.2 | 0.17 | 1.64 | 108.6 | 203.2 | 207.0 | 0.98 | 1.55 |
|  | P6 | 101.4 | 107.7 | 116.3 | 0.15 | 1.73 | 105.1 | 133.7 | 181.7 | 0.77 | 1.63 |
|  | P7 | 100.0 | 105.0 | 113.8 | 0.14 | 1.42 | 107.1 | 150.0 | 179.4 | 0.72 | 1.55 |
|  | P8 | 96.1 | 108.0 | 118.8 | 0.23 | 1.19 | 103.7 | 135.1 | 180.0 | 0.76 | 1.30 |
|  | P9 | 100.9 | 106.3 | 115.2 | 0.14 | 2.20 | 107.9 | 163.2 | 204.1 | 0.96 | 1.65 |
|  | P10 | 98.2 | 106.3 | 117.0 | 0.19 | 1.77 | 108.3 | 146.0 | 184.3 | 0.76 | 1.69 |
| SIB_54.4_ | P1 | 98.6 | 103.0 | 110.9 | 0.12 | 1.17 | 87.5 | 109.6 | 141.1 | 0.54 | 1.26 |
|  | P2 | 98.0 | 105.2 | 115.7 | 0.18 | 1.21 | 92.5 | 112.0 | 151.4 | 0.59 | 1.30 |
|  | P3 | 99.8 | 109.5 | 119.9 | 0.20 | 1.44 | 94.2 | 115.1 | 148.3 | 0.54 | 1.32 |
|  | P4 | 91.4 | 106.3 | 117.8 | 0.26 | 1.80 | 81.5 | 117.5 | 152.6 | 0.71 | 1.33 |
|  | P5 | 98.9 | 107.1 | 118.4 | 0.19 | 1.60 | 95.0 | 151.9 | 155.5 | 0.61 | 1.55 |
|  | P6 | 100.0 | 106.1 | 114.8 | 0.15 | 1.38 | 91.1 | 115.1 | 147.1 | 0.56 | 1.33 |
|  | P7 | 97.7 | 105.0 | 114.1 | 0.16 | 1.25 | 97.4 | 117.8 | 150.2 | 0.53 | 1.49 |
|  | P8 | 97.0 | 107.5 | 117.9 | 0.21 | 1.21 | 89.4 | 116.8 | 150.2 | 0.61 | 1.20 |
|  | P9 | 99.5 | 106.4 | 118.6 | 0.19 | 1.51 | 93.0 | 115.6 | 148.6 | 0.56 | 1.63 |
|  | P10 | 96.2 | 105.9 | 115.7 | 0.20 | 1.31 | 93.8 | 116.3 | 148.8 | 0.55 | 1.49 |
| SIB_48.0_ | P1 | 97.3 | 103.2 | 113.0 | 0.16 | 1.11 | 88.1 | 108.2 | 165.6 | 0.78 | 1.39 |
|  | P2 | 97.0 | 105.4 | 115.9 | 0.19 | 1.16 | 93.2 | 118.8 | 177.8 | 0.85 | 1.41 |
|  | P3 | 98.4 | 109.5 | 119.8 | 0.21 | 1.34 | 85.5 | 115.3 | 175.3 | 0.90 | 1.36 |
|  | P4 | 91.8 | 106.3 | 117.8 | 0.26 | 1.61 | 83.8 | 124.1 | 177.6 | 0.94 | 1.48 |
|  | P5 | 98.0 | 108.0 | 118.0 | 0.20 | 1.41 | 91.8 | 178.1 | 182.4 | 0.91 | 1.64 |
|  | P6 | 98.6 | 106.3 | 114.6 | 0.16 | 1.32 | 90.1 | 114.8 | 172.7 | 0.83 | 1.47 |
|  | P7 | 97.0 | 105.5 | 115.2 | 0.18 | 1.16 | 95.2 | 120.5 | 174.7 | 0.80 | 1.55 |
|  | P8 | 95.5 | 107.9 | 118.6 | 0.23 | 1.10 | 89.5 | 116.5 | 174.7 | 0.85 | 1.25 |
|  | P9 | 99.3 | 107.7 | 119.1 | 0.20 | 1.29 | 91.8 | 113.6 | 176.4 | 0.85 | 1.60 |
|  | P10 | 96.3 | 106.4 | 116.6 | 0.20 | 1.21 | 91.5 | 118.5 | 175.0 | 0.84 | 1.54 |

**Abbreviations:** CTV = clinical target volume; HR = high risk; LR = low risk; D% = dose to % of the CTV; HI = homogeneity index; CI = conformity index; SEQ = sequential boost; SIB = simultaneous integrated boost; P = patient.

**Supplementary Table S3.** Mean values (± 1 standard deviation) of the equivalent dose in 2-Gy(RBE) fractions (EQD2) obtained with the three treatment approaches, reported for both radiobiological calculation models. Statistically significant differences between the SEQ and SIB approaches are shown in bold (*p*-values are statistically significant after Bonferroni correction of 3 tests, *p* < 0.017.)

|  |  | HR-CTV | | | LR-CTV | | |
| --- | --- | --- | --- | --- | --- | --- | --- |
|  |  | EQD2(D98%) | EQD2(D50%) | EQD2(D2%) | EQD2(D98%) | EQD2(D50%) | EQD2(D2%) |
|  |  | [Gy(RBE)] | [Gy(RBE)] | [Gy(RBE)] | [Gy(RBE)] | [Gy(RBE)] | [Gy(RBE)] |
| LEM | SEQ | 89.1 ± 3.0 | 93.2 ± 0.0 | 95.3 ± 1.2 | 52.6 ± 3.3 | 81.4 ± 6.6 | 94.1 ± 0.8 |
|  | SIB_54.4_ | 87.7 ± 3.2 | 93.2 ± 0.0 | 95.6 ± 0.9 | **60.5 ± 3.3** | **76.2 ± 6.5** | 93.3 ± 1.2 |
|  | SIB_48.0_ | **86.5 ± 3.1** | 93.2 ± 0.0 | **96.1 ± 0.8** | 51.0 ± 1.8 | **68.6 ± 10.1** | 93.0 ± 1.7 |
| mMKM | SEQ | 71.4 ± 2.4 | 80.4 ± 2.1 | 93.8 ± 6.0 | 42.6 ± 2.9 | 67.5 ± 8.5 | 91.5 ± 3.8 |
|  | SIB_54.4_ | 70.3 ± 2.7 | 79.9 ± 2.0 | 92.2 ± 3.3 | 41.0 ± 2.9 | **60.5 ± 9.8** | **85.6 ± 3.4** |
|  | SIB_48.0_ | 69.4 ± 2.3 | 80.4 ± 2.0 | 92.8 ± 2.7 | **31.6 ± 1.7** | **49.9 ± 13.2** | **84.6 ± 3.2** |

**Abbreviations:** CTV = clinical target volume; HR = high risk; LR = low risk; LEM = local effect model; mMKM = modified microdosimetric kinetic model; SEQ = sequential boost; SIB = simultaneous integrated boost.
